# Toward a more nuanced understanding of probability estimation biases

**DOI:** 10.3389/fpsyg.2023.1132168

**Published:** 2023-03-30

**Authors:** Fallon Branch, Jay Hegdé

**Affiliations:** Department of Neuroscience and Regenerative Medicine, Medical College of Georgia, Augusta University, Augusta, GA, United States

**Keywords:** base rate neglect, cognitive rules of thumb, individuating information, inverse fallacy, judgement and decision-making under uncertainty, miss rate neglect, representativeness heuristic

## Abstract

In real life, we often have to make judgements under uncertainty. One such judgement task is estimating the probability of a given event based on uncertain evidence for the event, such as estimating the chances of actual fire when the fire alarm goes off. On the one hand, previous studies have shown that human subjects often significantly misestimate the probability in such cases. On the other hand, these studies have offered divergent explanations as to the exact causes of these judgment errors (or, synonymously, biases). For instance, different studies have attributed the errors to the neglect (or underweighting) of the prevalence (or base rate) of the given event, or the overweighting of the evidence for the individual event (‘individuating information’), etc. However, whether or to what extent any such explanation can fully account for the observed errors remains unclear. To help fill this gap, we studied the probability estimation performance of non-professional subjects under four different real-world problem scenarios: (i) Estimating the probability of cancer in a mammogram given the relevant evidence from a computer-aided cancer detection system, (ii) estimating the probability of drunkenness based on breathalyzer evidence, and (iii & iv) estimating the probability of an enemy sniper based on two different sets of evidence from a drone reconnaissance system. In each case, we quantitatively characterized the contributions of the various potential explanatory variables to the subjects’ probability judgements. We found that while the various explanatory variables together accounted for about 30 to 45% of the overall variance of the subjects’ responses depending on the problem scenario, no single factor was sufficient to account for more than 53% of the explainable variance (or about 16 to 24% of the overall variance), let alone all of it. Further analyses of the explained variance revealed the surprising fact that no single factor accounted for significantly more than its ‘fair share’ of the variance. Taken together, our results demonstrate quantitatively that it is statistically untenable to attribute the errors of probabilistic judgement to any single cause, including base rate neglect. A more nuanced and unifying explanation would be that the actual biases reflect a weighted combination of multiple contributing factors, the exact mix of which depends on the particular problem scenario.

## Introduction

In everyday life, ordinary people and trained professionals alike often encounter situations where they must estimate the probability of an event using imperfect evidence for the event. If the lawn is wet in the morning, what are chances that it rained during the previous night (Pearl, 1988)? What is the probability that there is an intruder in your yard if the dog barks? If someone is positively identified in a police lineup, how likely is it that this person is the actual culprit? What are the chances that the patient actually has cancer when a physician diagnoses one? Obviously, errors in estimating these probabilities can have significant real-world consequences.

A large number of previous studies have examined how well human subjects solve this problem in a wide variety of contexts (Kahneman and Tversky, 1973; Eddy, 1982; Kahneman et al., 1982; Fischhoff and Bar-Hillel, 1984; Thompson and Schumann, 1987; Bar-Hillel, 1991; Koehler, 1996; Villejoubert and David, 2002; Raacke, 2005; Kalinowski et al., 2008; Mandel, 2014; Raab and Gigerenzer, 2015; Dahlman et al., 2016). While these studies understandably vary in the exact task they used, they typically have the following design: The subjects are presented with a problem scenario, including the actual binary outcome (e.g., a patient is positively diagnosed with cancer or not) and the three underlying probabilistic factors: (i) true positive rate of the diagnosis, i.e., the probability that the patient actually has cancer given a positive diagnosis, (ii) false positive rate, the patient does not actually have cancer, and the diagnosis was a ‘false alarm’, and (iii) the prevalence, or base rate, of cancer in the given patient population. The subjects are then asked to estimate the actual probability of the outcome given the evidence for the outcome (e.g., probability that the patient actually has cancer given the diagnosis). The studies then compare the subjects’ probability estimates with the corresponding theoretically expected probabilities (see General Methods below for technical details).

Using this general approach, previous studies have consistently found that human subjects substantially misestimate the probabilities. That is, the subjects’ estimates typically deviate substantially from the theoretically expected probabilities (Eddy, 1982; Kahneman et al., 1982; Koehler, 1996; Mandel, 2014; Raab and Gigerenzer, 2015). Actually, for most real-world scenarios where the base rate is low, the subjects tend to *overestimate* the probability (Eddy, 1982; Kahneman et al., 1982; Koehler, 1996; Mandel, 2014; Raab and Gigerenzer, 2015).

An obvious next question is why. About this, previous studies have offered widely differing explanations: One longstanding view has been that these errors arise because the subjects attach too little weight to (or ‘underweight’, or neglect) the underlying prevalence, or base rate, of the event (Kahneman and Tversky, 1973; Fischhoff and Bar-Hillel, 1984; Bar-Hillel, 1991). This is why these judgements have been referred to as base rate fallacy, base rate neglect, or base rate bias (Kahneman and Tversky, 1973; Fischhoff and Bar-Hillel, 1984; Thompson and Schumann, 1987; Koehler, 1996; Dahlman et al., 2016). Some studies have also attributed the judgement errors to *overweighting* (i.e., attaching too much importance to) the evidence for a given individual event (or ‘individuating’ information; Kahneman and Tversky, 1973; Bar-Hillel, 1980; Kahneman and Tversky, 1982); the inverse fallacy (Villejoubert and David, 2002; Raacke, 2005; Kalinowski et al., 2008); and the so-called ‘miss rate neglect’, which actually refers to the neglect of false positive rates (Dahlman et al., 2016). On the one hand, few studies have explicitly claimed that any of these individual causes fully account for all of the observed errors. For instance, even those studies that attribute the estimation errors to base rate neglect stop short of explicitly offering base rate neglect as the *sole* explanation. On the other hand, it remains unclear as to whether and to what extent base rate neglect or any other aforementioned cause can, by itself fully account for the empirically observed errors.

The present study seeks to help fill this gap by focusing on a simple, straightforward question: When subjects estimate the probability of an event using the aforementioned established task paradigm, how much do various predictor variables contribute to the subjects’ estimated probabilities? We addressed this question using multiple different problem scenarios, and replicated the aforementioned biases in each case. We then quantitatively evaluated the extent to which the various potential causes contributed to the observed biases in each case. While we make no claims that our findings are the final word on this topic (see Discussion), we do show that there are principled reasons to call into question the prevailing explanations of what causes the observed biases.

## General methods

### Participants

The present study consisted of four mutually independent experiments. All procedures used in each experiment were approved in advance by the Institutional Review Board (IRB) of Augusta University, Augusta, GA, United States, where the experiments were carried out. Subjects were recruited using IRB-approved ads posted on various campus sites. All the subjects who participated in this research were adult volunteers with normal or corrected-to-normal vision, and provided informed consent prior to participating in the study. All were non-professional subjects, in the sense that none of the subjects had any known expertise in the task used in any of the four experiments, and that no subject was recruited, included, or excluded based on their education, training, or expertise. A total of 23 different subjects (mean age, 22.23 years ±4.23 [SD], excluding one subject whose age was not available; 16 women and one non-binary person) participated in this study. Some subjects participated in more than one experiment (see Supplementary material).

### Procedure

As noted above, accurately judging the probability of an actual outcome or event *A* (e.g., actual cancer) given binary evidence *B* for the event (e.g., diagnosis of cancer) requires one to jointly evaluate the following four pieces of information:

1. The prevalence, or base rate *p*(*A*) of the event,
2. The true positive rate, i.e., hit rate or *p*(*B|A*), which denotes the probability of observing the evidence. *B* given that event *A* has actually occurred,
3. The false positive rate, i.e., false alarm rate, *p*(*B|-A*), which denotes the probability of observing the evidence *B* given that event *A* has not actually occurred, and
4. Whether or not the evidence indicates the event has occurred, i.e.*, B* = 1 or *B* = 0. Given the aforementioned four pieces of information, the expected probability of the event *A* given that the evidence for the event had been observed, i.e., *B* = 1, is precisely specified by the Bayesian formula

(1a) p(A|B)=[p(A)p(B|A)]/[p(A)p(B|A)+p(−A)p(B|−A)].

The expected probability that the underlying event has not occurred given that evidence for the event has not been observed, i.e.*, B* = 0, is given by


(1b) $$\begin{array}{c} p\left(-A|-B\right)=\left[p\left(-A\right)p\left(-B|-A\right)\right]\\ /\left[p\left(A\right)p\left(B|A\right)+p\left(-A\right)p\left(B|-A\right)\right]. \end{array}$$

We used the above equations to calculate the theoretically expected probability for each given combination of input values for the equations (Eddy, 1982; Raab and Gigerenzer, 2015). It is important to emphasize, however, that our study neither required the subjects to estimate the probabilities in this fashion, nor did it assume that they did. That is, our study neither required the subjects to carry out mathematical calculations in their heads, nor assumed that this is how subjects do the task at hand.

Because the present study aimed to characterize the factors that underlie previously reported errors in probability estimation, we needed to reproduce the underlying errors in our study. For this reason, we simply adopted the task paradigm used in the influential study by Eddy (2005) and many others since [for a review, see Koehler, 1996]. Note that this study did not aim to, nor does it claim to, address the so-called ‘ecological validity’ of this task paradigm (Spellman, 1996).

### Task paradigm

During each trial, subjects were simultaneously given the above four items of information on a computer screen. For instance, in the context of Experiment 1 below, *p*(*A*) was the base rate of breast cancer; *p*(*B|A*) and *p*(*B|-A*) were the hit and false-alarm rates of a hypothetical CAD (computer-assisted diagnosis) system, and *B* was the binary decision of the system (see the Methods under the individual experiments below for details).

The meaning of each term was explained to the subjects interactively using both written and verbal explanations. We interactively ascertained that the subjects accurately understood the meanings of the terms prior to proceeding with the trials. Subjects were not provided any information whatsoever about the expected probabilities or approaches, Bayesian or otherwise, to carrying out the task.

Using only the information provided, subjects had to estimate, using a mouse-driven on-screen slider, the percent chance that the given event had actually occurred (also see individual experiments below). Subjects were afforded *ad libitum* opportunity to view the on-screen information and enter their response. They received no feedback.

The various rates and probabilities were presented both as fractions of 1 (e.g., 0.005) and as the corresponding ‘natural’ frequencies (e.g., 5 in 1000). This is because previous studies (Hoffrage and Gigerenzer, 1998; Hoffrage et al., 2015), and our preliminary work (Sevilla and Hegdé, 2017), have shown that some subjects are more comfortable with natural frequencies. Before the actual data collection, subjects underwent practice trials until they indicated they were fully familiar with all aspects of the task. The data from the practice trials were discarded.

### Data analysis

We analyzed the data using scripts custom-written in the R language (R_Core_Team, 2019). We carried out parametric statistical tests of significance where appropriate, and randomization-based tests of significance (Manly, 2007) otherwise. Where necessary, we corrected for multiple comparisons using the false discovery rate (FDR) method (Benjamini and Hochberg, 1995).

### Power analyses

These analyses were carried out using the R library *pwr*. Before initiating the present study, we carried out *a priori* power analyses to determine the subject recruitment target. To do this, we used the empirically observed fit of the data from a pilot study (Branch et al., 2022) as the expected fit of the model (see below), and calculated the total number of trials (pooled across all subjects). The results indicated that at least 63 trials (pooled across all subjects and repetitions) would be needed to achieve a statistical power of 0.90. *A posteriori* power analyses using the actual data indicated that our data achieved a power of >0.95 for the regression analyses in each of the four experiments in this study.

### Generalized linear mixed modeling

We used GLMM to determine the contribution of the various predictor variables to the subjects’ reported probabilities. GLMM is the appropriate modeling approach when the predictor variables are ‘mixed’, in that one or more variables are factorial or categorical (e.g., the binary decision of the system, in our case), and others are continuous (e.g., base rate; Dean and Nielsen, 2007; Berridge and Crouchley, 2011; Fox and Fox, 2016). GLMM has been used extensively for this purpose in psychological research (Dean and Nielsen, 2007; Berridge and Crouchley, 2011; Fox and Fox, 2016; Bono et al., 2021). In this report, we follow the recommended practices of reporting GLMM results [Bono et al., 2021; also see Cooper and American Psychological Association (2018)].

We carried out GLMM in two stages. We first constructed an exploratory model, which we will refer to as the “Initial Model,” in which we included as predictor variables all the primary independent variables in the given experiment and their pairwise interactions. For Experiments 1 through 3, the primary independent variables were base rate, false alarm rate, and the binary decision of the system. Hit rate was not included as a variable, because the hit rate was not varied in these experiments. The hit rate was varied in Experiment 4, and was included in the modeling of the results for Experiment 4.

Our modeling approach was designed to safeguard against the common pitfalls of regression modeling of real-world data (Aggarwal and Ranganathan, 2017; Ranganathan and Aggarwal, 2018). We will note many of the features of our approach in this section, and will highlight additional ones in context in the Results section of various experiments as appropriate, and will discuss the limitations of our approach in the General Discussion section.

One of the potential pitfalls of GLMM in particular, and of multiple regression in general, arises when the predictor (or independent) variables are mutually correlated, i.e., the nominally independent variables are not actually independent (Aggarwal and Ranganathan, 2017; Ranganathan and Aggarwal, 2018). Note that this caveat does not apply to our experiments, because the predictor variables were truly independent in that they were varied independently of each other. Note also that the fact that two or more predictor variables may have a joint influence on the response variable is not the same as the predictor variables being mutually correlated (Jaccard and Turrisi, 2003). In our models, such joint influences are captured by the interaction between predictor variables (Jaccard and Turrisi, 2003).

### Analysis of the relative importance of the independent variables: *lmg* statistic

The relative importance of predictor variables was assessed using the standard *lmg* statistic (Lindeman et al., 1980; Grömping, 2006). This is a well-established statistical analysis that can better assess the relative importance (or, equivalently, the relative contribution) of the predictor variables better than the conventional linear regression metrics, e.g., when the predictor variables covary (Lindeman et al., 1980; Grömping, 2006).

### Model selection

We used standard model selection procedures (Draper and Smith, 1998; Burnham et al., 2002) to evaluate the aforementioned Initial Model to determine the most parsimonious version of this model that accounted for greatest possible amount of the information in the data. Model selection is the standard approach to minimizing overfitting effects, one of the common pitfalls of multiple regression (Hegdé, 2021).

We will refer to the model ultimately selected in this fashion as the “Final Model.” Specifically, we used the aforementioned Initial Model as the input to a stepwise model selection algorithm that used the Akaike Information Criterion or AIC (Venables and Ripley, 2003). While model selection was carried independently of the aforementioned *lmg* analysis (and vice versa), the results of the two analyses were largely consistent with each other (not shown).

Note that the above modeling procedures make no assumptions about how the subjects arrived at their probability estimations. Note, in particular, that our models do not, however indirectly, utilize Equations 1a and 1b above. Instead, our models are data driven, our methods simply determine the model that best fits the empirical data at hand. Note also that GLMM modeling neither assumes nor requires that the underlying relationship between the predictor variables on the one hand and the response variables on the other is linear (Dean and Nielsen, 2007; Berridge and Crouchley, 2011; Fox and Fox, 2016). On the other hand, the GLMM approach does make certain standard assumptions about the nature of the underlying data (Dean and Nielsen, 2007; Berridge and Crouchley, 2011; Fox and Fox, 2016). In general, data in all four experiments adequately met these assumptions (data not shown). In particular, the residuals were normally distributed in all four experiments (not shown), indicating that the linear models adequately captured the underlying relationship between the independent variables vs. response variables (Fox and Fox, 2016; Fox and Weisberg, 2019).

### Relative contribution index

We calculated RCI values individually for each of the variables retained in the Final Model. We defined RCI value for the given variable *i* as


(2) $$\mathrm{RCI}=lmg_{i,actual}/lmg_{i,random}$$

where *lmg _i, actual_* was the actual *lmg* value for the given variable.

To calculate the *lmg _i, random_* value, we randomly reshuffled the values of each variable *i* across trials. We then refitted the same model to the randomized data and re-calculated the *lmg* value for each variable *i*. We repeated this process 1,000 times, calculated the *lmg* value for each variable *i*. The mean *lmg* value for a given variable *i* across the randomization was defined as the *lmg _i, random_* value for that variable. The uncorrected 95% confidence interval (CI) was defined as the 5^th^ and the 95^th^ percentiles the 1,000 *lmg _i, random_* values. The *p* value for the corresponding one-tailed alternative hypothesis was defined as the proportion of times the *lmg _i, random_* value was higher (or lower) than the *lmg _i, actual_* value (Manly, 2007). These *p* values were corrected for multiple comparison using the FDR method (Benjamini and Hochberg, 1995).

Note that the above RCI analysis implicitly uses the null hypothesis that all the predictor variables contribute equally to the observed probability estimates and tests this hypothesis against the empirical data. This is a principled approach, especially because the aforementioned previous studies of neglect implicitly assume that the proper estimation requires equal weighting (Kahneman and Tversky, 1973; Fischhoff and Bar-Hillel, 1984; Thompson and Schumann, 1987; Koehler, 1996; Dahlman et al., 2016).

## Experiment 1: Estimating the probability of cancer in a mammogram based on CAD system evidence

### Methods

Thirteen subjects (10 women; mean age, 19.67 years ±1.67) participated in this experiment. Subjects were simultaneously given four items of information on a computer screen:

1. The prevalence, or base rate, of breast cancer in the given cohort of patients [i.e., *p*(*A*) in Eqs. 1a,b above],
2. The hit rate *p*(*B|A*) of a hypothetical CAD system for breast cancer detection,
3. The false alarm rate *p*(*B|-A*) of the system, and
4. The binary decision of the system as to whether or not a given mammogram was positive for cancer. No mammogram was shown. That is, the subjects had to estimate the probability that the given unseen mammogram was positive for cancer based solely on the above four items of information.

During this Experiment, we held the hit rate constant at 1.0, and systematically varied the remaining three variables, and measured its effect on the subjects’ estimated probabilities of cancer. During any given trial, values for each of the three variables were randomly drawn from the corresponding repertoire of possible values: two possible values of the base rate (0.05 or 0.005), five possible values of the false alarm rate (0.05, 0.25, 0.5, 0.75 and 0.95), and two possible values for the binary decision of the CAD system (0 or 1, corresponding to whether the mammogram was positive or negative for cancer, respectively). Note that the values of the four variables varied independently from one trial to the next. Each possible combination of these values was tested exactly once during each block of 20 trials. Subjects performed 1 or 2 blocks each. Data were pooled across subjects.

It is worth noting that the data we present in this experiment are entirely independent of the data we have presented in a comparable previous study that was designed to address a different issue (Branch et al., 2022). That is, the data in the two studies were collected independently of each other using non-overlapping sets of subjects. Moreover, task parameters used in the previous study were different from those used in this experiment.

### Results

**Figure 1:**
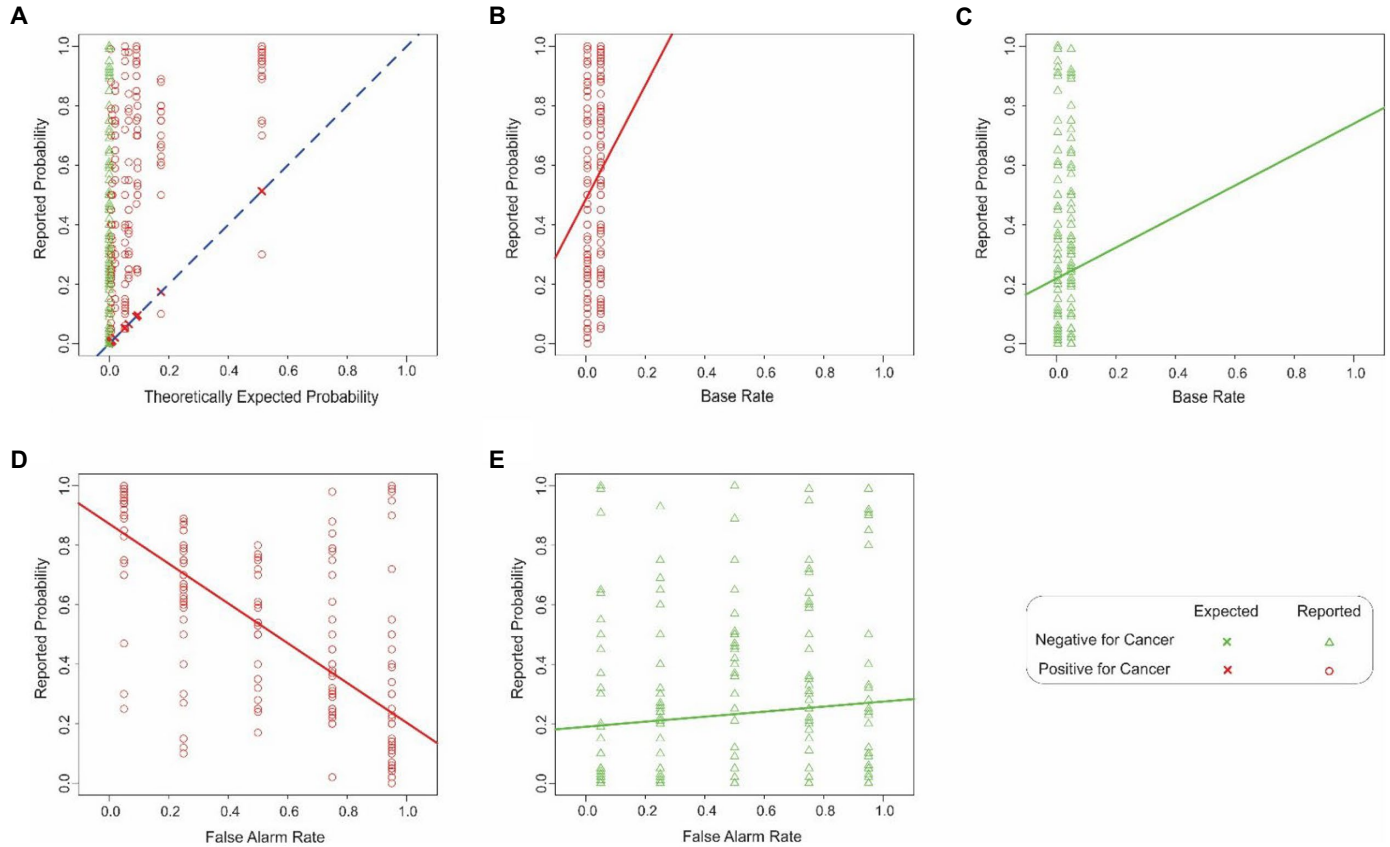
Estimation errors in Experiment 1. **(A)** Probability of cancer estimated by the subjects (*y-axis*) as a function of the corresponding theoretically expected probabilities (*x*-axis). Each *red circle* or *green triangle* denotes a single trial in which the hypothetical CAD system decided that the mammogram in question was positive or negative for cancer, respectively (see *legend* at *bottom right*). The *‘X’* symbols and the *dashed diagonal* denote hypothetical scenarios where the subjects’ estimated probability exactly matched the corresponding expected probability. The color of the plotting symbols (*red* vs. *green*) denote individual trials in which the CAD system determined that the given mammogram was positive or negative for cancer, respectively. The lines denote the best-fitting linear regression line in each case. **(B,C)** The interaction between the base rate and the binary decision of the system. The same plotting conventions as in panel A are used, except that in this panel, the estimated probability (*y-axis*) is plotted against the base rate (*x-axis*). For visual clarity, the data corresponding to the two decisions of the CAD system (mammogram positive or negative for cancer) are shown separately in panel d and e, respectively. In either panel, the *solid* and *dashed lines* denote best-fitting regression line. Panels **(D,E)** similarly show the interaction between the false alarm rate and the binary decision of the system, the estimated probability (*y-axis*) is plotted as a function of whether the CAD system decided that the mammogram was positive or negative for cancer (panel **D** or **E**), respectively. See text for details.

The cancer probability estimates pooled across all subjects are plotted as a function of the corresponding theoretically expected probabilities in Figure 1A, where each plotting symbol denotes the reported probability estimate from an individual subject during a single trial (see legend for details). The plotting symbols corresponding to the two decisions of the CAD system (i.e., that the given mammogram is positive or negative for cancer) are denoted as a red circle or green triangle, respectively (see key at bottom right of Figure 1). Each vertical column represents the data points for a single theoretically expected probability.

Two qualitative aspects of these results are worth noting. First, the subjects generally misestimated the probability of cancer, as denoted by the fact that the estimates (*red circles* and *green triangles*) differed substantially from the theoretically expected probabilities (‘*X*’ symbols and the *diagonal*). If the subjects had estimated the probability correctly, all their estimates would overlap the X symbol in the given column. Instead, the subjects’ estimates deviated substantially from the theoretically correct estimates. Across all subjects, the maximum and minimum difference between the reported vs. expected percent probabilities were 1.0 and − 0.21, respectively. The average difference was 0.33 ± 0.29 (standard deviation).

Second, the estimated values typically were *overestimates*, as denoted by the fact that most of the estimates were above the diagonal. The overestimates were highly significant (1-tailed paired *t*-test, *t* = 26.60, *df* = 519, *p* < 2.2^−16^). This systematic bias straightforwardly indicates that the subjects failed to estimate the probabilities accurately. Intriguingly, the subjects’ overestimates were significantly larger when the mammogram was deemed positive for cancer than when they were deemed negative (1-tailed *t*-test, *t* = 8.61, *df* = 516.15, *p* < 2.2^−16^). Together, these results suggest that the subjects were performing the task intuitively, rather than using systematic, logical reasoning.

To help quantify the extent to which the various explanatory (or predictor) variables contributed to the subjects’ estimates, we constructed a generalized linear mixed model (GLMM), in which we included all three independent variables we varied in this experiment, along with their pairwise interactions as predictors (see Methods for details). This exploratory model (or ‘Initial Model’) is summarized in Table 1. We report the results about both the beta (or regression) coefficients β_i_ (columns A – D in Table 1) and the coefficients of determination *R^2^* (column E) of this model, because they both provide useful, but mutually distinct, types of information about the underlying data, as briefly outlined below.

**Table 1:** Summary of regression modeling of the reported probabilities in Experiment 1.

Predictor variable in the initial model^‡^		Exploratory linear regression model				*lmg* value (% contribution to overall *R*^2^)^†^*
		Estimated coefficient *β*	Standard error	*t* value	*p* value	
**#**	Name	A	B	C	D	E
1	Null model (intercept only)	0.20	0.04	5.19	2.99 × 10^−7^	(N.A.)
2	Base rate of cancer in the cohort	−3.39	0.99	−3.39	0.69	2%
3	False alarm rate of the CAD system	0.03	0.06	0.57	0.57	19%
4	Binary decision of the system	0.64	0.05	13.75	<2 × 10^−16^	48%
5	Interaction of base rate & false alarm rate	1.82	1.45	1.25	0.21	0.4%
6	Interaction of base rate & binary decision	1.39	0.94	1.48	0.14	0.5%
7	Interaction of false alarm rate & binary decision	−0.75	0.07	−11.54	<2 × 10^−16^	31%

^‡^See Methods for additional details. ^†^The model as a whole accounted for 45.59% of the variance (i.e., *R*^2^ = 0.4559).

* Model selection procedures retained variables # 2, 3, 4, 6, and 7 in the Final Model (not shown).

The Initial Model is given by the relationship


(3) $$\hat{y}=\beta_{0}+\beta_{1}x_{1}+\beta_{2}x_{2}+\ldots +\beta_{7}x_{7}+\varepsilon$$

where *ŷ* is the model’s *estimates* of the values of the response variable (as opposed to the actual observed values *y* of the response variable); *x*_1_ through *x*_7_ are the seven predictor variables included in this model; β_1_ through β_7_ are the corresponding weight coefficients of the predictor variables; β_0_ is the model offset; and *ε* is the error, so that *ε* = *y* - *ŷ*. That is, the β values are scaling coefficients that collectively specify the offset (in case of β_0_) and the slope (in case of β_i_) of the regression line that best fits the data. They determine the values of the estimates *ŷ* directly as shown in Eq. 3, and are only indirectly related to actual observed values *y*. Thus, interpreting β values as representing the contribution of the predictor variables to the observed responses can be misleading to the extent to which *ŷ* differs from *y*, especially when the observed responses are scattered widely about the regression line (Draper and Smith, 1998; Burnham et al., 2002; Aggarwal and Ranganathan, 2017). On the other hand, to the extent to which *ŷ* is correlated with *y*, the best coefficients do provide useful information about the contribution of the predictor variables to the observed response. After all, beta coefficients are used for this purpose extensively in psychology, neuroscience, econometrics, etc. (Friston, 2007; Gravetter and Wallnau, 2017; Laha, 2019; Hashimzade and Thornton, 2021). Regression coefficients are also essential for model selection, i.e., for determining which predictor variable/s make a statistically significant contribution to *ŷ*, and therefore should be retained in the parsimonious ‘Final Model’ of the data (Draper and Smith, 1998; Burnham et al., 2002).

On the other hand, for the purposes of measuring the contribution of the various predictor variables to the observed responses, metrics that reflect the *statistical correlation* between *x* and *y* are more appropriate (Draper and Smith, 1998; Burnham et al., 2002). For this purpose, we use the well-established *lmg* statistic (column E, Table 1), which denotes the percent contribution of the given predictor variable to the observed responses [see General Methods for details; also see Lindeman et al. (1980)].

An examination of the Initial Model indicated that the base rate of cancer in the patient cohort made a statistically insignificant contribution to the model (row 2). This straightforwardly suggests that the subjects underweighted, i.e., neglected, the base rate in making their decisions.

As noted above, many previous studies have suggested that base rate neglect occurs because subjects not only underweight the base rate but also simultaneously attach too much importance to the ‘individuating information’, i.e., the binary decision of the system about the individual mammogram in the present case (Kahneman and Tversky, 1973). The contribution of the binary decision factor to the subject’s responses was indeed highly significant (row 4).

Note, however, the fact the binary decision *contributed significantly* does not necessarily mean that it *overcontributed*, i.e., that it contributed more than its share to the model. If, for the sake of argument, the subjects attached exactly correct weight to this factor (i.e., neither underweighted nor overweighted it), the contribution of this factor could still be statistically significant. Thus, statistically significant contribution does not necessarily mean overcontribution/overweighting. We will revisit this issue below using additional analyses.

The false alarm rate by itself did not make a statistically significant contribution to the model at the level of 95% confidence in this model (row 3). However, the interaction between the false alarm rate and the binary decision of the system did (row 7). That is, the false alarm rate affected the subjects’ reports differentially depending on the binary decision of the system. This interaction is reflected in the fact that the best-fitting regression lines are different in Figures 1B,C. In other words, the subjects’ estimates covaried with the false alarm rates when the CAD system decided that the individual mammogram was positive for cancer (Figure 1B), but not when the mammogram was deemed negative for cancer (Figure 1C), a finding confirmed by a 2-way analysis of covariance (ANCOVA; false alarm rate x binary decision; *p* < 0.05 for both factors and their interaction, not shown). It is also worth noting that the estimated coefficient of this interaction factor was negative (Estimated Coefficient = −0.75; row 7, column A of Table 1), indicating that the overall effect of this factor was to reduce the estimated probabilities. By contrast, the binary decision had an effect of a comparable magnitude, but of opposite sign (Estimated Coefficient 0.64; row 4, column A). Thus, the overall estimates of the responses reflect a complex interplay of multiple, sometimes counteracting, factors.

The coefficient of determination of the Initial Model, *R*^2^, was 0.4559, indicating that the seven predictor variables in this model collectively accounted for about 46% of the variance in the observed responses (see Footnote to Table 1). This raises the issue of how much each predictor variable contributed to this 45.59%. As noted above, previous studies have variously attributed such estimation errors to neglect or overweighting (i.e., where a given variable contributes less or more than its share) of the various underlying variables. Therefore, it is crucially important to determine the relative contribution of each of the variables in the present case.

To do this, we used the well-established method of the *lmg* index (Lindeman, Merenda and Gold index; Lindeman et al., 1980); *lmg* index (see Methods). The *lmg* index is a principled method for decomposing a given *R*^2^ value into the relative contributions from the various independent variables. It is equivalent to, but distinct from, partial *R*, and offers some advantages over the latter (Lindeman et al., 1980). Under the null hypothesis (i.e., default assumption) that all six variables contributed equally to the overall fit, i.e., that the subjects weighted each variable appropriately, each variable is expected to contribute 1/6 ≈ 16.67% to the *R^2^* value, i.e., explained variance or the model fit (see General Methods). The actual contributions are shown in column E of Table 1. The most important contributor to the subjects’ estimates was the individuating information, and it accounted for 48% of the *R*^2^ value (row 4, column E). Similarly, the false alarm-binary decision interaction and the false alarm rate, respectively, accounted for about 31% and 19% of the *R*^2^ value. Thus, the subjects nominally overweighted each of these three variables (also see below). On the other hand, subjects underweighted, or neglected, the remaining three variables (rows 2, 5, and 6, column E).

The above results are based on the Initial Model that included all seven of the original predictor variables. It is well known that including too few or too many predictor variables can lead to modeling artifacts (Draper and Smith, 1998; Fox and Fox, 2016; Hegdé, 2021); therefore, it is desirable to optimally balance model complexity with model fit (Draper and Smith, 1998; Fox and Fox, 2016), i.e., to determine the most parsimonious model that accounts for the most amount of observed data. We used standard model selection procedures to determine such a parsimonious model for this experiment, which we will refer to as the ‘Final Model’ [see Methods for details; also see Draper and Smith (1998) and Fox and Fox (2016)]. The Final Model retained just five predictor variables: (i) base rate, (ii) false alarm rate, (iii) binary decision, (iv) base rate-binary decision interaction, and (v) the false alarm-binary decision interaction, indicating that only these five factors had a statistically significant effect on the subjects’ estimates (rows 2, 3, 4, 6, and 7 in Table 1; also see footnote to Table 1).

The aforementioned *lmg* value analysis did not address whether or not the relative contributions of the various variables were statistically significant. For instance, the fact that base rate is retained in the Final Model as a significant predictor of the outcome is noteworthy, but does it mean that the subjects do not significantly neglect base rate at all, i.e., do they give base rate its due weight in arriving at their estimates?

**Figure 2:**
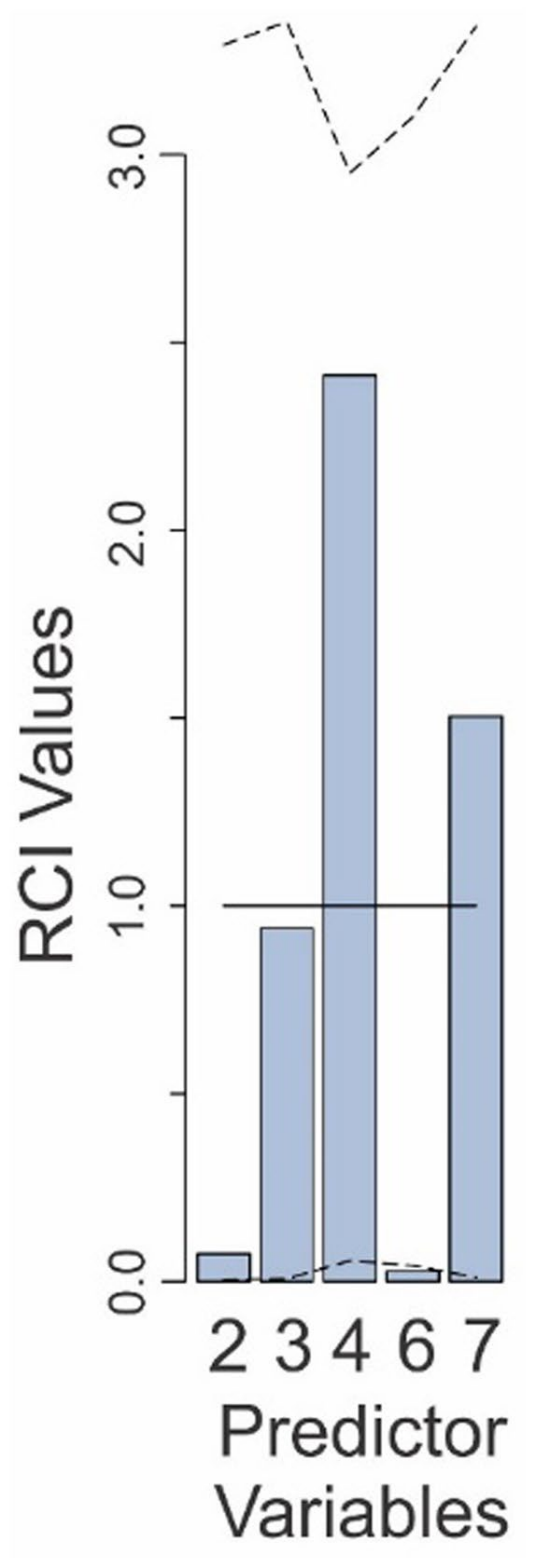
Relative contributions of various predictors to the fit of the Final Model in Experiment 1. The predictor variables are those that are retained in the Final Model and are numbered as in Table 1. The *solid line* denotes the expected contributions of the various predictors. The *dashed lines* denote the upper and lower 95% confidence intervals (uncorrected), empirically determined from the data. See text for details.

To help address such issues, we calculated the Relative Contribution Index (RCI) for each of the five predictors in the Final Model (see General Methods for details). The RCI value for a given predictor is essentially its *lmg* value adjusted for the level of randomness in the empirical data. That is, the RCI value of the predictor measured the extent to which the actual *lmg* value for a given predictor compares to the *lmg* value for that predictor expected from random chance (see General Methods for details), where a value of 1.0 indicated that the predictor contributed exactly the expected amount to the outcome, and values >1 and < 1, respectively, indicate correspondingly higher or lower contribution than the contribution expected for that predictor. The RCI values for the five predictors in the Final Model are shown in Figure 2.

The RCI value for the base rate factor was 0.07 (predictor 2 in Figure 2), well below the RCI value expected from random (*solid line* in Figure 2), indicating that the subjects indeed underweighted the base rate substantially. However, this RCI value was still within the 95% confidence interval (CI: 0.006–3.39; see *dashed lines* in Figure 2), indicating that the underweighting was not statistically significant at 95% confidence level.

On the other hand, the underweighting of the false alarm-binary decision interaction was indeed statistically significant (predictor 6; RCI = 0.027; CI: 0.042–3.1). The false alarm rate contributed slightly less than the expected amount (predictor 3; RCI = 0.94; CI: 0.007–3.35). The binary decision of the system, as well as the false alarm-binary decision interaction both made larger-than-average contributions to the outcome (RCI values of 2.4 and 1.5, respectively), although this was not statistically significant (CIs: 0.06–3.1 and 0.01–3.34, respectively). When results were corrected for multiple comparisons (see Methods), the contribution of none of the variables remained statistically significant (not shown). Collectively, these results show that while subjects substantially underweighted (or neglected) some variables and overweighted some others, while only the binary decision-dependent neglect of the base rate was statistically significant.

### Discussion

The above results show that naive subjects significantly overestimate the probability of cancer. They also identify multiple sources of these estimation errors, including the overweighting of some factors such as the binary decision of the CAD system, and underweighting other factors such as the base rate. In this regard, our results confirm and extend the previous studies to the present task.

These results are novel in three main respects. First, our results demonstrate that both underweighting and overweighting contribute to the estimation errors. Second, our results identify two additional contributing factors, namely the base rate-dependent neglect of false alarm rates, and the binary decision-dependent overweighting of the false-alarm rate. Previous studies have reported the neglect of false alarm rates (which the reports referred to as ‘miss rate neglect’) in the context of legal judgements (Dahlman et al., 2016; Dahlman and Mackor, 2019). But to our knowledge, our study is the first to report the contribution of the above two factors and to report such *conditional* underweighting/overweighting. Finally, we demonstrate that the underweighting or overweighting of *individual* factors is not statistically significant although the collective effect of all the factors together is a significant overestimation of cancer probabilities, as noted above.

Our preliminary studies indicate that highly trained, practicing radiologists also commit similar errors in the same task (Branch et al., 2022). Thus, overestimation of the probabilities was not attributable to the fact that the subjects in the present experiment were untrained professionals.

## Experiment 2: Estimating the probability of drunkenness based on breathalyzer evidence

The results of Experiment 1 raise the issue of whether and to what extent they are idiosyncratic to the particular task that the subjects were carrying out. For instance, it may be that subjects tended to overestimate the probability of chance because of the perceived costs of underestimating the cancer risk. To the extent this is true, the pattern of estimation errors would change if the same problem was posed in a different problem context where costs of various types of errors (e.g., false positives and false negatives) were different. We tested this hypothesis in the present experiment by keeping all the parameters exactly the same, but using them to pose a different problem, namely estimating the probability of drunk driving based on the outcome of individual breathalyzer tests.

### Methods

This experiment was identical to Experiment 1 except for the task. In this experiment, the subjects were told that the four items of information pertained to a breathalyzer system that was used for testing motorists for drunk driving. Specifically, the four parameters were:

1. The base rate of drunk driving in the given cohort of motorists,
2. The hit rate of a hypothetical breathalyzer system,
3. The false alarm rate of the system, and
4. The binary decision of the system (positive or negative for drunkenness) for a given motorist from the given cohort of motorists. No other data were provided to the subjects. Twelve subjects (eight women; mean age, 19.58 years ±1.44) participated in this experiment.

### Results

**Figure 3:**
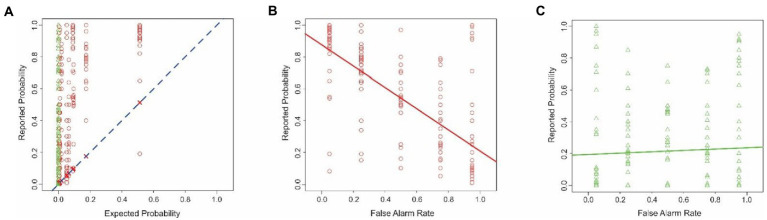
Estimation errors in Experiment 2. The data are plotted according to the conventions used in Figure 1. **(A)** Probability of drunk driving estimated by the subjects plotted as a function of the corresponding theoretically expected probability. **(B,C)** Interaction between the false alarm rate and the binary decision of the breathalyzer. See text for details.

The reported probabilities in this experiment (Figure 3A) were collectively indistinguishable from the results in Experiment 1 (two-tailed *t*-test, *p* > 0.05; not shown), indicating that changing the task did not result in large-scale changes in the reported probabilities overall. The subjects’ reported estimates deviated substantially from the theoretically expected probabilities (Figure 3A). Across all subjects, the maximum and minimum difference between the reported vs. expected percent probabilities were 1.0 and − 0.32, respectively. The average difference was 0.33 ± 0.30. The subjects also significantly overestimated the probabilities (1-tailed paired *t*-test, *t* = 23.47, *df* = 459, *p* < 2.2^−16^). Also, magnitude of the overestimations was significantly larger when the mammogram was deemed positive for cancer than when it was deemed negative (one-tailed *t*-test, *t* = 8.69, *df* = 457.61, *p* < 2.2^−16^).

With the exception of the base rate-binary decision interaction, all of the predictors that contributed significantly to the outcome in Experiment 1 also did so in this experiment. The nature of the false alarm-binary decision interaction was similar to that in Experiment 1, so that the subjects took the false alarm rate into account when the breathalyzer system determined that the motorist was drunk, but not when the system decided otherwise (Figures 3B,C; ANCOVA; false alarm rate x binary decision; *p* < 0.05 for both factors and their interaction, not shown). This interaction and the binary decision variable made a significant contribution to the outcome in the Initial Model (Table 2, rows 4 and 7, column D). These two variables and two additional variables, including the base rate and the false alarm rate, were retained in the Final Model (see footnote to Table 2).

**Table 2:** Summary of regression modeling of the reported probabilities in Experiment 2.

Predictor variable in the initial model^‡^		Exploratory linear regression model				*lmg* value (% contribution to overall *R*^2^)^†^*
		Estimated coefficient *β*	Standard error	*t* value	*p* value	
**#**	**Name**	A	B	C	D	E
1	Null model (intercept only)	0.15	0.04	3.53	4.6 × 10^−4^	(N.A.)
2	Base rate of drunk driving in the cohort	1.57	1.08	1.45	0.15	0.6%
3	False alarm rate of the breathalyzer system	0.05	0.07	0.77	0.44	20%
4	Binary decision of the system	0.72	0.05	13.91	<2 × 10^−16^	53%
5	Interaction of base rate & false alarm rate	−0.33	1.59	−0.21	0.83	0.01%
6	Interaction of base rate & binary decision	−1.19	1.04	−1.15	0.25	0.3%
7	Interaction of false alarm rate & binary decision	−0.71	0.07	−9.91	<2 × 10^−16^	26%

^‡^See Methods for additional details. ^†^The model as a whole accounted for 45.28% of the variance (i.e., *R*^2^ = 0.4528).

* Model selection procedures retained variables # 2, 3, 4, and 7 in the Final Model (not shown).

**Figure 4:**
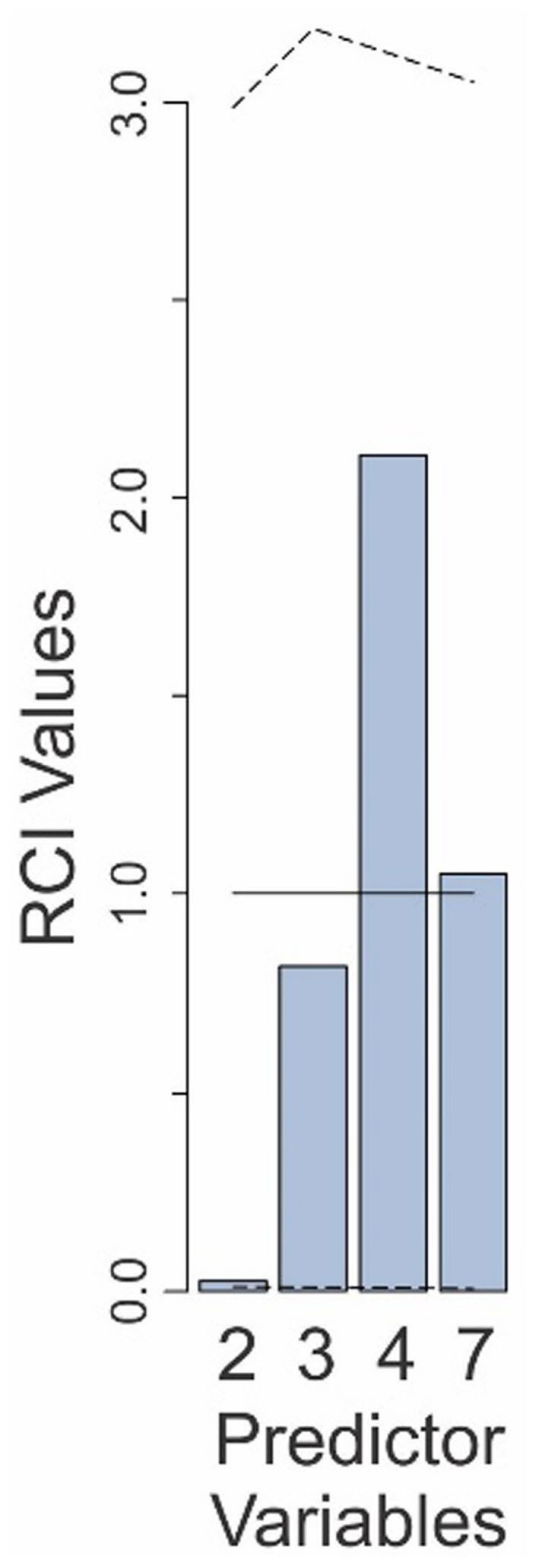
Relative contributions of various predictors to the fit of the Final Model in Experiment 2. The predictor variables are those that are retained in the Final Model and are numbered as in Table 2. The *solid line* denotes the expected contributions of the various predictors. The *dashed lines* denote the upper and lower 95% confidence intervals (uncorrected), empirically determined from the data. See text for details.

Results of the RCI analysis showed that all four factors retained in the Final Model contributed substantially to the final outcome, and the under/overweighting of none of the contributions was statistically significant, even without correction for multiple comparisons (Figure 4).

### Discussion

One notable difference between the results of this experiment from those in Experiment 1 was that the binary decision-base rate interaction was retained in the final mode in Experiment 1, but not in this experiment. Other than that, the results of this experiment were similar to those of Experiment 1. Most notably, our analyses showed no evidence for significant neglect or overweighting of any other variables in the present experiment, either. These results indicate that changing the task had little or no effect on the estimation of probabilities.

## Experiment 3: Estimating the probability of an enemy sniper based on evidence from drone reconnaissance system

### Methods

This experiment was identical to Experiments 1 and 2, except for the task. In this experiment, the subjects were told that the four items of information pertained to a military drone system that was used to reconnoiter a combat scene for enemy snipers. Specifically, the four parameters were:

1. The prevalence of enemy snipers in the given theater of combat operations,
2. The hit rate of the drone system,
3. The false alarm rate of the system, and
4. The binary decision of the system (positive or negative for the presence of an enemy sniper) for a given combat scene from the given theater of operations. No other data were provided to the subjects. The subjects had to estimate the probability that an enemy sniper was present in the scene of combat. Thirteen subjects (nine women; mean age, 20.23 years ±2.39) participated in this experiment.

### Results and discussion

**Figure 5:**
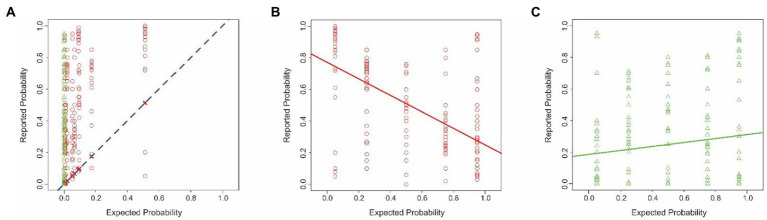
Estimation errors in Experiment 3. The data are plotted according to the conventions used in Figure 1. **(A)** Probability of enemy sniper estimated by the subjects is plotted here as a function of the corresponding theoretically expected probability. **(B,C)** Interaction between the false alarm rate and the binary decision of the reconnaissance drone. See text for details.

The subjects’ responses in this experiment (Figure 5A) were indistinguishable from the results in Experiment 1 by a two-tailed *t*-test, *p* > 0.05; not shown.

The subjects’ reported estimates deviated substantially from the theoretically expected probabilities (Figure 5A). Across all subjects, the maximum and minimum difference between the reported vs. expected percent probabilities were 0.95 and − 0.46, respectively. The average difference was 0.33 ± 0.29. The subjects significantly overestimated the probabilities (1-tailed paired *t*-test, *t* = 23.55, *df* = 419, *p* < 2.2^−16^). This systematic bias straightforwardly indicates that the subjects failed to estimate the probabilities accurately. Intriguingly, the subjects’ overestimates were significantly larger when the combat scene was deemed positive for enemy sniper than when it was deemed negative (one-tailed *t*-test, *t* = 5.94, *df* = 412.77, *p* = 3.08^−09^; also see Figures 5B,C).

**Figure 6:**
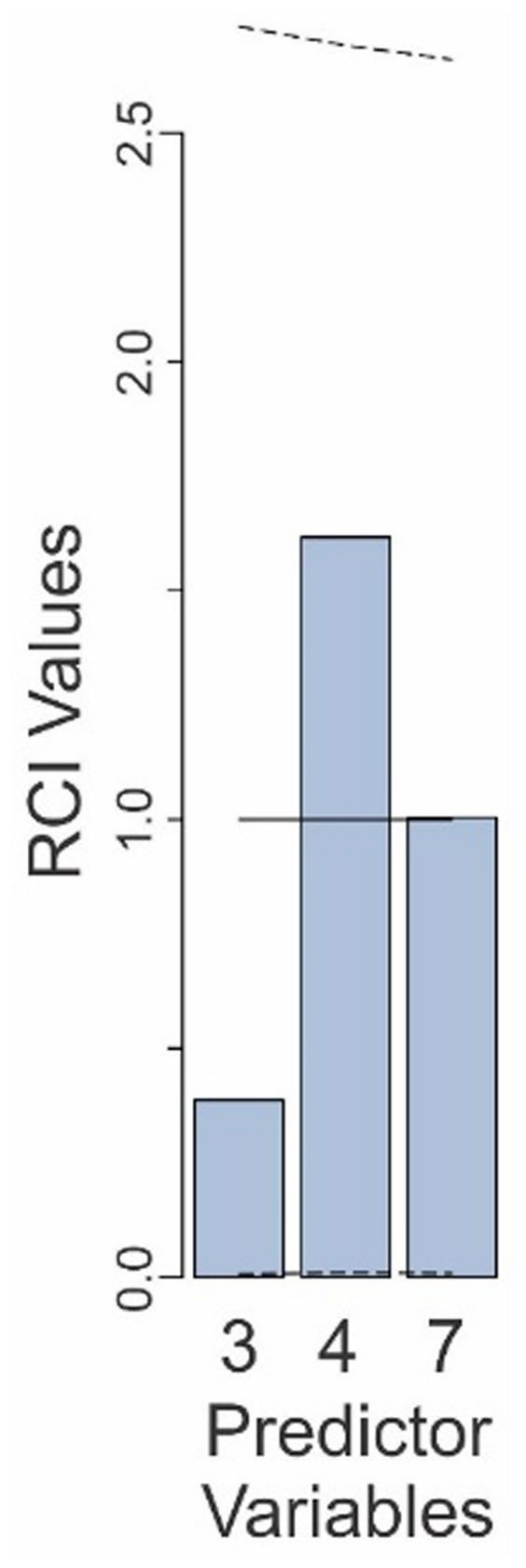
Relative contributions of various predictors to the fit of the Final Model in Experiment 3. The predictor variables are those that are retained in the Final Model and are numbered as in Table 3. The *solid line* denotes the expected contributions of the various predictors. The *dashed lines* denote the upper and lower 95% confidence intervals (uncorrected), empirically determined from the data. See text for details.

In this experiment, only three predictor variables were retained in the Final Model: false alarm rate of the drone system, binary decision of the system, and the false alarm-binary decision interaction (see footnote to Table 3). Recall that all three variables were also retained in Experiments 1 and 2, but two additional predictors were retained in those experiments that were not retained in this experiment, raising the possibility that the variables in question were over/underweighted in the present experiment. However, the over/underweighting of none of the variables was statistically significant in this experiment, even without correction for multiple comparisons (Figure 6).

**Table 3:** Summary of regression modeling of the reported probabilities in Experiment 3.

Predictor variable in the initial model^‡^		Exploratory linear regression model				*lmg* value (% contribution to overall *R*^2^)^†^*
		Estimated coefficient *β*	Standard error	*t* value	*p* value	
**#**	**Name**	A	B	C	D	E
1	Null model (intercept only)	0.16	0.05	3.46	5.9 × 10^−4^	(N.A.)
2	Base rate (i.e., prevalence of snipers in the given theater of combat)	0.87	1.19	0.73	0.46	0.8%
3	False alarm rate of the reconnaissance drone system	0.14	0.07	1.88	0.06	13%
4	Binary decision of the system	0.58	0.06	10.31	<2 × 10^−16^	52%
5	Interaction of base rate & false alarm rate	−0.46	1.75	−0.26	0.79	0.04%
6	Interaction of base rate & binary decision	0.14	1.14	0.13	0.90	0.008%
7	Interaction of false alarm rate & binary decision	−0.65	0.08	−8.23	2.51 × 10^−15^	34%

^‡^See Methods for additional details. ^†^The model as a whole accounted for 32.55% of the variance (i.e., *R*^2^ = 0.3255).

* Model selection procedures (not shown) retained variables # 3, 4, and 7 in the Final Model (not shown).

## Experiment 4: Estimating the probability of an enemy sniper based on evidence from drone reconnaissance system (version 2)

In Experiments 1–3, only the problem scenario differed across the experiments, but numerical values of the four probabilistic parameters remained the same. This design helped us address the important issue of the extent to which the estimation errors vary or remain the same depending on the problem scenario. The present experiment took the complementary approach of varying the parameter values while keeping the problem scenario unchanged.

This tweak in the experimental design allowed us to test additional hypotheses about the underlying phenomenon. For instance, subjects in Experiments 1–3 showed a conditional neglect of the false alarm rate, wherein subjects underweighted the false alarm rate differently based on the binary decision of the system. The present experiment was designed to test the hypothesis that the subjects show a similar conditional neglect of the *hit rate.* A second hypothesis is that all other things being equal, subjects attach more weight to the hit rate than to the false alarm rate.

### Methods

This experiment was identical to Experiment 3, except in two respects: To help better characterize the effect of varying the false alarm rates, we increased the number of possible hit rates to three (0.05, 0.5, and 0.95), so that the hit rate during any given trial was randomly drawn from these three values. Second, the false alarm rate during any given trial was drawn from the palette of the same three values (i.e., 0.0, 0.05, 0.5, and 0.95). As alluded to above, the problem scenario remained the same as in Experiment 3, so that the subjects estimated the probability that an enemy sniper was present in the scene of combat. Seven subjects (five women and one non-binary person; mean age, 27.71 years ±2.43) participated in this experiment.

### Results and discussion

**Figure 7:**
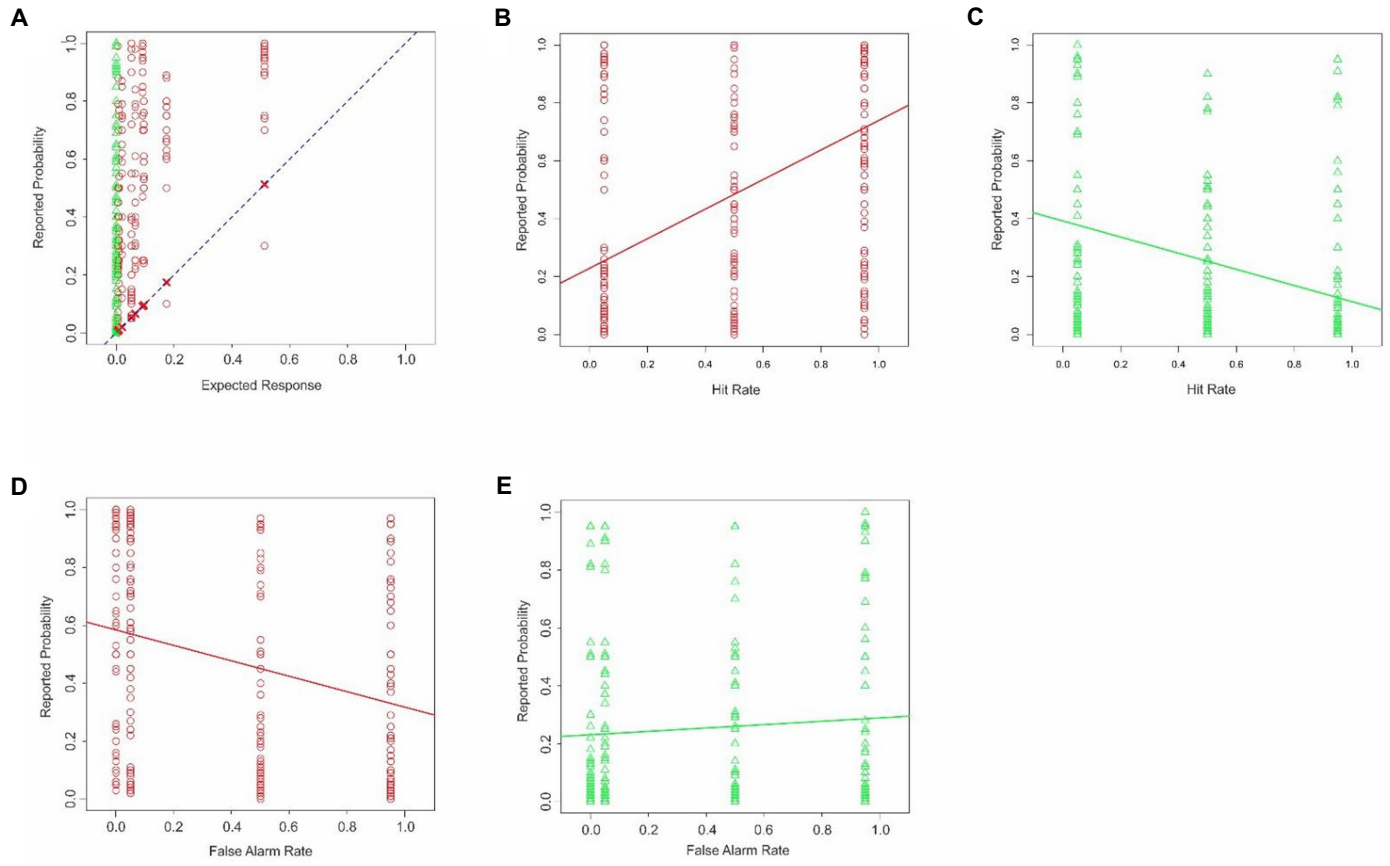
Estimation errors in Experiment 4. The data are plotted according to the conventions used in Figure 1. **(A)** Probability of enemy sniper estimated by subjects is plotted in this Figure as a function of the corresponding theoretically expected probability. **(B,C)** Interaction between the hit rate and the binary decision of the reconnaissance drone. **(D,E)** Interaction between the false alarm rate and the binary decision of the drone. See text for details.

The reported probabilities in this experiment (Figure 7A) were collectively indistinguishable from the results in Experiment 1 (two-tailed *t*-test, *p* > 0.05; not shown), indicating that changing the task did not result in large-scale changes in the reported probabilities overall. The subjects’ reported estimates deviated substantially from the theoretically expected probabilities (Figure 7A). Across all subjects, the maximum and minimum difference between the reported vs. expected percent probabilities were 0.96 and − 0.97, respectively. The average difference was 0.19 ± 0.40. The subjects significantly overestimated the probabilities (1-tailed paired *t*-test, *t* = 14.81, *df* = 1,007, *p* < 2.2^−16^). This systematic bias straightforwardly indicates that the subjects failed to estimate the probabilities accurately. However, in contrast to the results obtained in Experiments 1–3, the subjects’ overestimates were statistically indistinguishable between the combat scene was deemed positive for enemy sniper than when it was deemed negative (one-tailed *t*-test, *t* = −0.59, *df* = 890.22, *p* = 0.72).

As noted above, unlike in Experiments 1–3, the hit rate was varied in this experiment. This manipulation revealed a new interaction, namely the conditional neglect of hit rates, wherein the subjects underweighted the hit rate of the drone system based on the binary decision of the system (Figures 7B,C; ANCOVA; hit rate x binary decision; *p* < 0.05 for both factors and their interaction, not shown). The subjects also showed a conditional neglect of the false alarm rate (Figures 7D,E; ANCOVA; false alarm rate x binary decision; *p* < 0.05 for both factors and their interaction, not shown).

In this experiment, six different predictor variables were retained in the Final Model: base rate, hit rate, false alarm rate, binary decision of the system, and two interaction factors: the hit-rate binary decision interaction and the false alarm rate-binary decision (rows 2–7 in Table 4; also see footnote to Table 4). The two factors involving hit rate retained in this experiment were not available in Experiments 1–3.

**Table 4:** Summary of regression modeling of the reported probabilities in Experiment 4.

Predictor variable in the initial model^‡^		Exploratory linear regression model				*lmg* value (% Contribution to overall *R*^2^)^†^*
		Estimated coefficient *β*	Standard error	*t* value	*p* value	
**#**	**Name**	A	B	C	D	E
1	Null model (intercept only)	0.37	0.04	9.74	<2 × 10^−16^	(N.A.)
2	Base rate (i.e., prevalence of snipers in the theater of combat)	−0.01	0.92	−0.01	0.99	0.05%
3	Hit rate of the reconnaissance drone system	−0.28	0.05	−5.11	3.90 × 10^−07^	4.34%
4	False alarm rate of the system	0.03	0.06	0.59	0.55	3.9%
5	Binary decision of the system	−0.05	0.04	−1.10	0.27	32%
6	Interaction of hit rate & binary decision	0.79	0.05	15.39	<2 × 10^−16^	50%
7	Interaction of false alarm rate & binary decision	−0.33	0.05	−6.64	5.14 × 10^−11^	9.37%
8	Interaction of hit rate & base rate	−0.30	1.14	−0.26	0.80	0.02%
9	Interaction of false alarm rate & base rate	0.62	1.09	0.57	0.57	0.07%
10	Interaction of base rate & binary decision	0.24	0.84	0.29	0.77	0.02%
11	Interaction of false alarm rate & hit rate	0.02	0.07	0.23	0.82	0.01%

^‡^See Methods for additional details. ^†^The model as a whole accounted for 32.06% of the variance (i.e., *R*^2^ = 0.3206).

* Model selection procedures retained variables # 2, 3, 4, 5, 6, and 7 in the Final Model (not shown).

**Figure 8:**
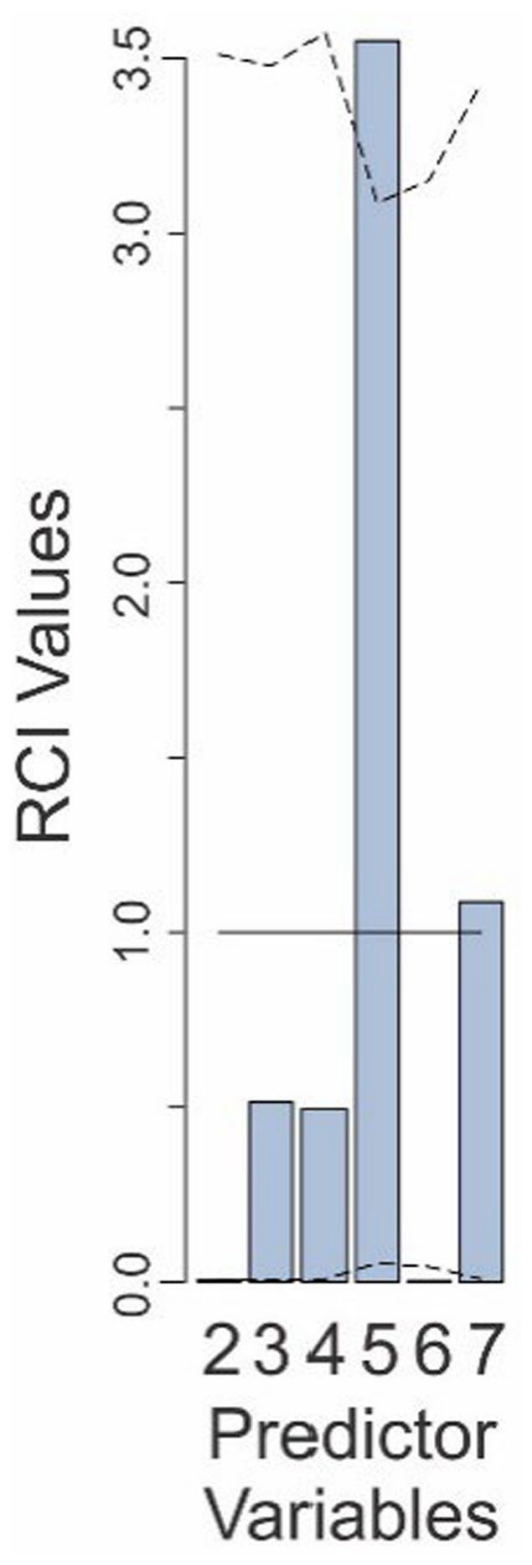
Relative contributions of various predictors to the fit of the Final Model in Experiment 4. The predictor variables are those that are retained in the Final Model and are numbered as in Table 4. The *solid line* denotes the expected contributions of the various predictors. The *dashed lines* denote the upper and lower 95% confidence intervals (uncorrected), empirically determined from the data. See text for details.

RCI analysis (Figure 8) showed that, of the six factors retained in the Final Model in this experiment, the relative contribution of only two—binary decision and the hit rate-binary decision interaction (predictors 5 and 6, respectively)—were significantly outside the uncorrected 95% confidence intervals. However, only the hit rate-binary decision interaction factor survived the correction for multiple comparisons, indicating that the relative contribution of this factor was significantly smaller than expected. That is, judging by the RCI analysis, this factor can be reasonably deemed to be significantly neglected. That is, the subjects’ failure to properly weight the individuating information as a function of the hit rate was statistically significant.

While one may be tempted to claim that the hit rate-binary decision interaction factor in this experiment was the only factor in our entire study to be significantly over/underweighted, doing so would be unwise. This is because making this comparison would require correction for this extended multiple comparison, in which this factor does not survive.

## General discussion

### Generalizability of probability estimation errors

Several aspects of the estimation errors were common to all four experiments in our study. First of all, subjects failed to make accurate judgements in each experiment. Second, the judgement errors were large, and varied widely from the theoretically expected estimations. Finally, the estimation errors represented significant overestimations in all four experiments.

It is also noteworthy that the overall pattern of errors was statistically indistinguishable across the four experiments (one-way ANOVA; *p =* 0.98 for the between-experiment factor; data not shown), even though the tasks and/or underlying probabilistic parameters varied across the experiments. This indicates that the errors were a general feature of the estimation problem used in our study, and generalized across the tasks and the experimental parameters we used. This straightforwardly suggests that the subjects are unlikely to have used grossly different mental strategies for estimating the probability of the outcome.

### Factors that contribute significantly to estimation errors

Our analyses identified multiple contributing factors for the errors. Both the similarities and differences among these factors across experiments are noteworthy. On the one hand, factors such as overweighting of the binary decision (i.e., individuating information) and the underweighting (or neglect) of the base rate were major contributing factors to the errors across all four experiments. These findings are consistent with the large body of earlier studies using this task paradigm as well as other task paradigms that have attributed the errors variously to one or both of these factors (Kahneman and Tversky, 1973; Fischhoff and Bar-Hillel, 1984; Thompson and Schumann, 1987; Koehler, 1996; Baratgin and Noveck, 2000; Fantino, 2004; Barbey and Sloman, 2007; Dahlman et al., 2016; Sanborn and Chater, 2016; also see Koehler (1996) and the accompanying commentaries).

On the other hand, some factors made statistically significant contributions to the outcome in some experiments and not others. For instance, the interaction between the base rate and binary decision was evident in Experiments 1 and 4, but not in the other two experiments. Further studies are needed to address the issue of why exactly the relative contributions of factors differed across tasks.

Our study also identified several additional contributing factors that, to our knowledge, have not been previously reported. The most notable among these are the factor interactions. We identified many statistically significant interactions across the experiments (Tables 1–4). Of particular note is the interaction between false alarm rates and binary decisions, whereby the subjects attach different weight to the false alarm rates depending on the binary decision (and vice versa). Intriguingly, this interaction was statistically significant in all four of the experiments. To our knowledge, such interaction (or, ‘conditional’) effects have not been reported before, although previous studies have reported a neglect of the false alarm rates (sometimes referred to as the “miss rate neglect”) in the context of legal decision-making (Thompson and Schumann, 1987; Dahlman et al., 2016).

### Causes of the errors are significant as a group, not individually

Our results show that the aforementioned factors, as a group, do account for a significant amount of the subjects’ estimates of the probabilities. Depending on the experiment, the independent variables collectively account for about 30 to 45% of the variance, depending on the experiment. Of course, this is unsurprising, because in any study, the independent variables would be expected to account for the response variable/s, to the extent that the former have any bearing on the latter.

What is surprising about our results is the fact that, by a principled set of criteria, none of the contributing factors by itself significantly accounts for the outcome (see below for caveats). As noted earlier, many previous studies have attributed these errors variously to the neglect of base rates, overweighting of the evidence for the individual event, or both [for an overview, see Koehler (1996) and the accompanying commentaries]. The collective effect of these studies has been substantial, in that the estimation errors in question have come to be widely known as the base rate neglect, base rate fallacy, or base rate bias (Kahneman et al., 1982; Gigerenzer and Hoffrage, 1995; Fantino, 2004). Some previous studies have attributed these errors in other contexts, such as legal decision-making, to the so-called fallacy of the transposed conditional or the prosecutor’s fallacy, where the subjects conflate *p*(*A*|*B*) for *p*(*B*|*A*) (Thompson and Schumann, 1987), or to the neglect of false alarm rates, sometimes referred to as the miss rate neglect (Dahlman et al., 2016).

While these studies provide empirical evidence that subjects do underweight (or conflate, in case of the prosecutor’s fallacy) the relevant variables, they do not show that these factors by themselves fully account for the errors. In fairness to such studies, few of them expressly claim that factors such as base rate neglect fully account for the errors. However, factors such as base rate neglect have somehow come to be thought of as sufficient explanations for the underlying errors.

Our study successfully reproduces the estimation errors, and demonstrates that such claims are misleading at best, because they obscure the complexities of the underlying phenomena. On the one hand, our results unambiguously show that subjects make large, systematic errors, which straightforwardly means that the subjects fail to correctly weight the various underlying factors to one degree or another. This in turn raises the question of what level of underweighting constitutes neglect. For instance, if the subject underweights the base rate factor by, say, an average of 10%, can this legitimately be deemed base rate neglect? Previous studies have generally avoided this issue. This study takes the position that underweighting can be deemed neglect if it is statistically significant, i.e., if the weight is significantly lower than that expected from random chance. Similarly, a given factor can be considered overweighted if it is significantly larger than that expected from random chance. These clearly are principled criteria, but by no means the only possible ones (see below).

### Some important caveats

In addition to the various methodological caveats noted in context throughout this report, a few caveats are especially worth highlighting here: First, as alluded to above, our study focused narrowly on the question of whether and to what extent the observed biases can be accounted for by the overweighting or neglect of *individual* factors, as implied by the earlier studies. For this reason, our study remained advisedly agnostic about a variety of important, vigorously debated questions in the field. Chief among these are issues such as (i) how the subjects arrive at their estimates (Kahneman et al., 1982; Koehler, 1996), (ii) approaches to reducing the estimation errors and efficacy of these errors (Hoffrage and Gigerenzer, 1998; Uhlmann et al., 2007; Raab and Gigerenzer, 2015), (iii) the methodological and conceptual validity and usefulness of formulating and studying the probability estimations within the Bayesian framework (Koehler, 1996; Baratgin and Noveck, 2000; Fantino, 2004; Barbey and Sloman, 2007; Sanborn and Chater, 2016), and (iv) whether and to what extent our findings generalize to other task paradigms of probability estimation (e.g., Gigerenzer, 1996; Koehler, 1996), or when tested using a larger number of disparate problem scenarios. Further studies are needed to address each of these questions.

In addition to the various methodological caveats noted in context throughout this report, two caveats are especially worth highlighting here: First, as its name indicates, GLMM assumes a *linear* relationship between the predictor variables and the response variable. While our GLMMs did indeed satisfy the underlying assumptions (data not shown), this does not by itself prove that the actual underlying relationship is linear. Indeed, it remains possible that there exists an unknown non-linear relationship that accounts for the observed data even better.

## Concluding remarks

A main significance of our study is that it calls into question the validity of attributing the probability estimation errors to individual factors. But in a larger sense, the significance of our study is that it proposes a set of reasonable criteria and methods for evaluating the potential causes of probability estimation errors.

## Data availability statement

The original contributions presented in the study are included in the article/Supplementary material, further inquiries can be directed to the corresponding author.

## Ethics statement

The studies involving human participants were reviewed and approved by Institutional Review Board (IRB) of Augusta University, Augusta, GA, United States. The patients/participants provided their written informed consent to participate in this study.

## Author contributions

FB and JH jointly designed the study, collected and analyzed the data, and wrote the manuscript. All authors contributed to the article and approved the submitted version.

## Funding

This study was supported by grant # W911NF-15-1-0311 from the Army Research Office (ARO) to JH.

## Conflict of interest

The authors declare that the research was conducted in the absence of any commercial or financial relationships that could be construed as a potential conflict of interest.

## Publisher’s note

All claims expressed in this article are solely those of the authors and do not necessarily represent those of their affiliated organizations, or those of the publisher, the editors and the reviewers. Any product that may be evaluated in this article, or claim that may be made by its manufacturer, is not guaranteed or endorsed by the publisher.
