# Supplementary material for: Toward a more nuanced understanding of probability estimation biases
Source: Front Psychol. 2023 Mar 30;14:1132168. doi: 10.3389/fpsyg.2023.1132168 (PMC10101207; doi:10.3389/fpsyg.2023.1132168)
Supplement: Supplementary file 1 [file Table_1.pdf]

Supplementary Material

Toward A More Nuanced Understanding of Probability Estimation Biases

Fallon Branch[1] and Jay Hegdé[1]*

[1] Department of Neuroscience and Regenerative Medicine, Medical College of Georgia, Augusta University, Augusta, GA, USA

*Corresponding Author [E-Mail: jhegde@augusta.edu]

Table 1. Demographic profile of the subjects in this study

Participant	Demographic Characteristic		Experiment			
#	Gender*	Age (yrs)	Exp 1	Exp 2	Exp 3	Exp 4
17	F	22	X			
45	F	20	X			
46	F	18	X	X	X	
47	F	19		X		
48	M	20		X	X	
49	F	19	X	X	X	
04	M	18	X		X	
50	M	20	X	X		
51	F	18	X	X	X	
52	F	20	X	X	X	
53	M	20		X	X	
54	M	23	X	X	X	
55	F	19	X	X	X	
00	F	18	X	X	X	
56	F	Between 18 to 65 §	X			
01	F	21	X	X	X	
57	F	25			X	X
58	F	24			X	X
59	F	28				X
01	M	28				X
60	F	29				X
61	NB	29				X
62	F	31				X

X = The subject participated in this Experiment
* Self-identified. F = female; M = male; NB = non-binary
§ Best available information. This person’s age was excluded from age calculations.
